# Plant-Derived B-CGT Hydrogel Accelerates Diabetic Wound Healing Through Multitarget Modulation of Inflammation, Angiogenesis, and Tissue Remodeling

**DOI:** 10.3390/gels11020104

**Published:** 2025-02-02

**Authors:** Fei Ran, Kailang Mu, Lingli Zhou, Leqiang Peng, Gang Liu, Yuchen Liu, Yuxin Pang, Guo Feng, Changmao Guo, Tianjian Wang, Qiumei Luo

**Affiliations:** College of Pharmacy, Guizhou University of Traditional Chinese Medicine, Guiyang 550025, China; rf991125@163.com (F.R.); mkl980818@163.com (K.M.); pyxmarx@126.com (Y.P.);

**Keywords:** diabetic wound healing, B-CGT hydrogel, mechanism of action, network pharmacology, molecular docking

## Abstract

Diabetic wound healing presents significant challenges due to impaired angiogenesis, chronic inflammation, and cellular dysfunction. Building on previous research, this study further explores the potential of a plant-derived glucosyloxybenzyl 2-isobutylmalates (B-CGT) hydrogel in promoting diabetic wound healing. Network pharmacology and molecular docking analyses suggest that B-CGT may regulate key mechanisms, such as apoptosis, inflammation, and matrix remodeling, through core targets including SIRT1, CASP8, and MMP8. In vivo studies further demonstrated that B-CGT hydrogel significantly accelerated wound closure in diabetic mice, enhanced angiogenesis, promoted collagen deposition, and achieved immune balance by modulating macrophage polarization, thereby shifting the inflammatory environment toward a repair state. Moreover, B-CGT hydrogel significantly improved the wound microenvironment by upregulating VEGF expression and exerting antioxidant effects. By combining theoretical predictions with experimental validation, this study elucidates the multi-target synergistic regulatory mechanisms of B-CGT hydrogel. These findings provide new research directions for addressing immune imbalance and angiogenesis defects in diabetic wound healing and lay a scientific foundation for the optimization and application of chronic wound treatment strategies.

## 1. Introduction

Diabetes is a chronic metabolic disease with a global prevalence, characterized by prolonged hyperglycemia. This condition not only directly impairs the function of pancreatic β-cells but also leads to multi-organ damage [1,2,3]. Diabetes significantly disrupts the daily life of patients and triggers a series of complex complications, among which diabetic wounds, especially chronic diabetic ulcers, have become a major medical challenge worldwide [4,5,6]. Due to prolonged hyperglycemia, diabetic patients often suffer from multiple pathological changes, including neuropathy, vascular abnormalities, and immune suppression, all of which severely affect normal wound healing [6,7,8,9,10].

The healing process of diabetic wounds is typically delayed, with recurrent inflammation and infection, significantly increasing the disease burden on patients [11,12,13]. In chronic wounds of diabetic patients, the wound frequently enters a state of chronic inflammation. This prolonged phase not only exacerbates local tissue necrosis but also hinders the regeneration process. Several studies have indicated that one of the major barriers to wound healing in diabetic patients is the disruption of the local immune response and the impaired ability of the tissue to transition from inflammation to repair [10,14,15,16].

In addition to immune dysregulation, diabetes also impairs local microcirculation, further exacerbating wound healing issues [17,18]. Chronic hyperglycemia causes endothelial cell damage and impairs angiogenesis, limiting the supply of oxygen and nutrients to the wound, thereby reducing its regenerative capacity [4,19,20,21]. Diabetic patients often exhibit insufficient angiogenesis, slow granulation tissue formation, and abnormal collagen deposition during wound healing, which contribute to the occurrence and progression of chronic wounds [6,22,23].

The healing process of acute wounds, in contrast, typically follows a defined sequence of stages and occurs relatively quickly in healthy individuals [10,24,25]. Acute wounds, such as cuts or abrasions, are caused by sudden physical injury, and the body usually responds with rapid tissue repair. Unlike diabetic wounds, acute wounds do not face the same persistent inflammation or impaired angiogenesis [21,26]. In healthy individuals, acute wound healing is marked by an efficient transition from inflammation to tissue proliferation and remodeling, ultimately restoring the tissue to its normal function without prolonged complications [24,25,27,28].

Thus, addressing immune dysregulation and impaired angiogenesis in diabetic wound healing has become a central issue in wound therapy. While some clinical treatments, such as dressing changes, debridement, and antibiotic application, can improve the local environment of diabetic wounds to a certain extent, these traditional methods still face significant limitations, especially in promoting wound healing [29,30,31]. Therefore, the search for novel, efficient therapeutic strategies has become a key focus in modern medical research.

In recent years, biomaterials, especially hydrogels, have shown great promise in wound healing. Due to their unique physicochemical properties, such as excellent biocompatibility, good hydration, and adjustable drug release characteristics, hydrogels have been widely applied in chronic wound therapy [32,33,34]. The three-dimensional network structure of hydrogels, which closely resembles the extracellular matrix (ECM), provides an ideal growth environment for cells, promoting their proliferation, adhesion, and migration [35]. Thus, hydrogels not only provide an ideal protective layer for wounds but also locally deliver drugs or growth factors to modulate the wound environment and accelerate healing.

Against this backdrop, we propose using a hydrogel based on a natural plant extract, B-CGT (glucosyloxybenzyl 2-isobutylmalates from Bletilla striata) hydrogel, aimed at leveraging its unique biological activity to promote diabetic wound healing. Preliminary studies have shown that B-CGT hydrogel possesses multiple biological functions, including antioxidant, anti-inflammatory, and cell proliferation-promoting effects. In vitro, B-CGT hydrogel demonstrated a good ability to promote proliferation, adhesion, and migration of fibroblasts. In animal experiments, B-CGT hydrogel significantly improved wound healing outcomes by accelerating angiogenesis and promoting fibroblast proliferation [36,37].

Given the favorable performance of B-CGT hydrogel in conventional wound healing, this study further investigates its role and mechanism in the healing of chronic diabetic wounds. A full-thickness diabetic mouse wound model was established to evaluate the efficacy of B-CGT hydrogel in diabetic wound healing and to systematically explore its underlying mechanisms. First, network pharmacology screening was performed to predict the potential molecular targets of B-CGT and their roles in signaling pathways. Subsequently, molecular docking analysis was performed to validate the binding affinity between B-CGT and key targets, providing support for its molecular mechanism. Finally, in vivo experiments were carried out to assess the biological effects of B-CGT hydrogel in promoting diabetic wound healing, including immunomodulation, angiogenesis, and tissue remodeling. We hypothesize that B-CGT hydrogel accelerates wound healing by modulating the immune microenvironment and correcting angiogenesis dysfunction in diabetic wounds through a multi-target, multi-pathway synergistic mechanism. This study not only offers new insights into the treatment of diabetic wounds but also provides scientific evidence for the development of multifunctional therapeutic materials based on natural products.

## 2. Results and Discussion

### 2.1. Network Pharmacology Study

#### 2.1.1. Prediction of B-CGT Targets and Intersection Analysis with Diabetic Wound Healing Targets

**Figure 1:**
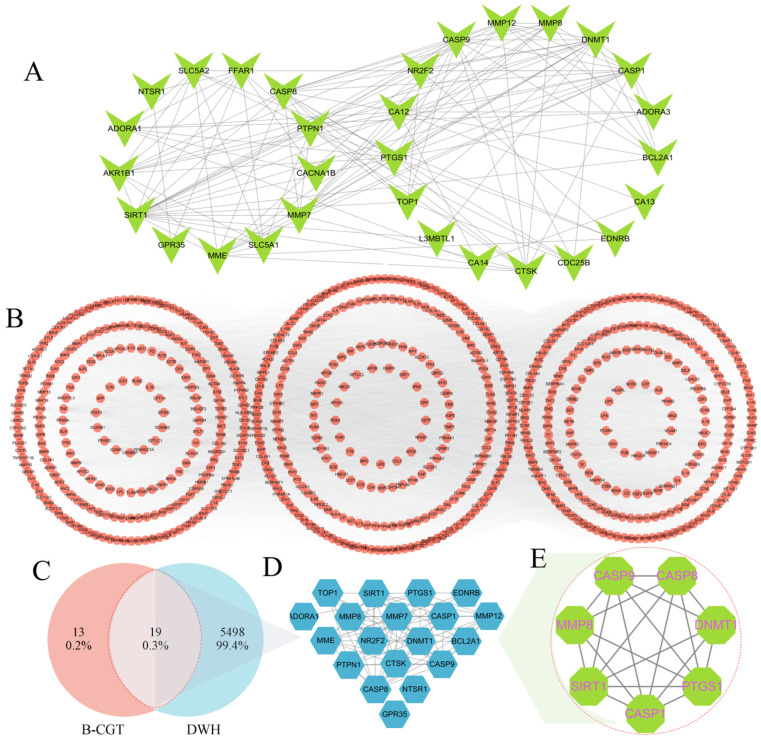
Network analysis of B-CGT drug targets, diabetic wound healing disease targets, and core target interactions. (**A**): Drug target network, including 32 potential drug targets. (**B**): Network of 811 disease targets with a score greater than 10. (**C**): Venn diagram showing the drug–disease intersecting targets. (**D**): Network diagram of 19 intersecting targets. (**E**): Final core target network, including seven core targets.

Target prediction of B-CGT was performed using the TargetNet online tool (filter criterion: AUC ≥ 0.7), which identified 32 potential targets (Table 1). These include key proteins such as PTPN1, SIRT1, and CASP8, which are involved in inflammation regulation, apoptosis, and diabetes. A drug-target network was constructed using Cytoscape 3.10.2 software (Figure 1A). Network topology analysis revealed that targets such as PTPN1 and SIRT1 had high centrality, suggesting they may play crucial roles in the biological activity of B-CGT. PTPN1, a key regulator of the insulin signaling pathway, is closely associated with the pathophysiology of diabetes, while SIRT1 plays an important role in anti-inflammatory and metabolic regulation. These findings suggest that B-CGT may improve diabetic wound healing by modulating multiple key targets [38,39].

To further elucidate the mechanism of B-CGT in diabetic wound healing, we retrieved 5517 disease-related targets from the GeneCards database (Figure 1B) and performed an intersection analysis with B-CGT’s drug targets. The results revealed 19 common targets (Figure 1C,D), which are likely to mediate the pharmacological effects of B-CGT in diabetic wound healing. Based on topology analysis using the CytoNCA plugin, and with criteria such as DC, BC, CC, EC, NC, and LAC all greater than the median value, seven core targets were selected from the intersecting targets. These core targets include SIRT1, CASP8, CASP9, CASP1, DNMT1, PTGS1, and MMP8 (Figure 1E; Table 2).

The biological functions of these core targets are closely related to diabetic wound healing. For example, SIRT1 improves diabetes-related complications by regulating cellular metabolism and anti-inflammatory processes [40]; CASP1, CASP8, and CASP9, as key nodes in the apoptosis pathway, may promote wound healing by regulating the release of inflammatory factors [41,42,43]; MMP8 is involved in wound tissue repair by modulating extracellular matrix degradation and remodeling [44,45].

#### 2.1.2. Functional Annotation and Enrichment Analysis

**Figure 2:**
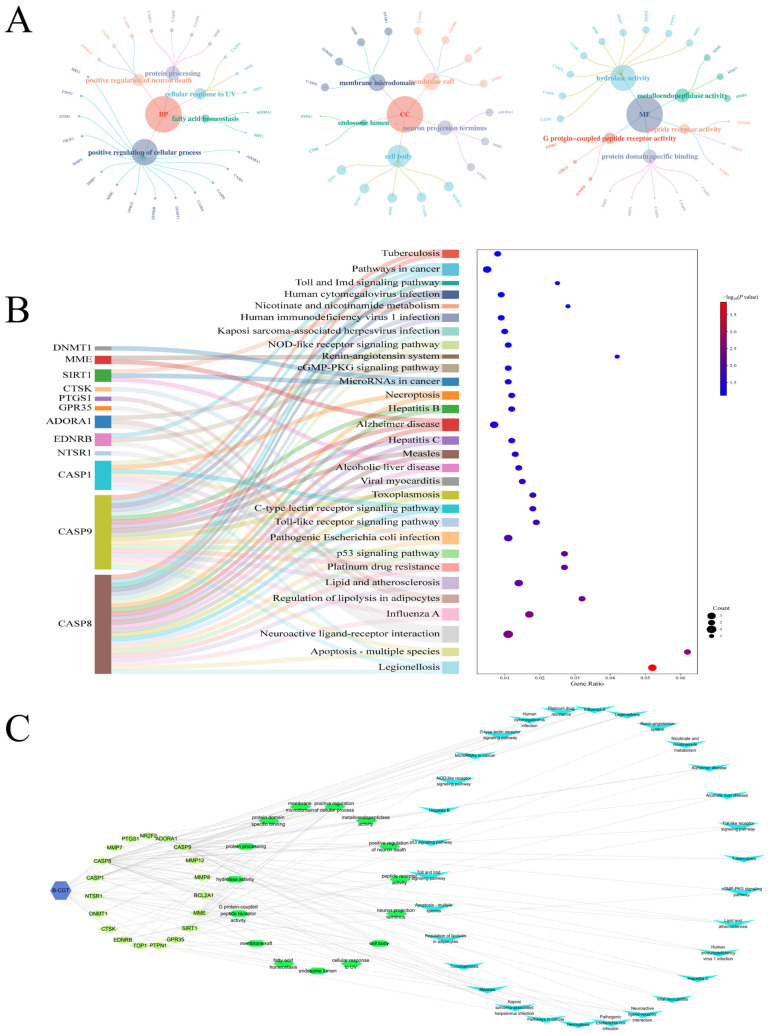
B-CGT drug-target-function-pathway network analysis. (**A**): GO functional enrichment of B-CGT and disease intersection targets (including cellular component, biological process, and molecular function); (**B**): KEGG pathway enrichment of B-CGT and disease intersection targets (including the top 30 pathways ranked by significance); (**C**): Integration and visualization of the drug-target-function-pathway network.

To further explore the mechanism of B-CGT, we conducted Gene Ontology (GO) functional annotation and a Kyoto Encyclopedia of Genes and Genomes (KEGG) pathway enrichment analysis on its intersecting targets. The GO analysis revealed significant enrichment of intersection targets at three levels: Cellular Component (CC), Biological Process (BP), and Molecular Function (MF) (Figure 2A, Table 3). At the CC level, the intersection targets were predominantly associated with “cell body” (GO:0044297) and “membrane raft” (GO:0045121). In the BP category, functions closely related to diabetic wound healing, such as “positive regulation of cell processes” (GO:0048522) and “fatty acid homeostasis” (GO:0055089), were significantly enriched. In terms of MF, functions like “hydrolase activity” (GO:0016787) and “metalloendopeptidase activity” (GO:0004222), both relevant to wound repair and inflammation regulation, were notably enriched.

KEGG pathway analysis further identified potential pathways associated with the intersection targets (Figure 2B, Table 4). Significantly enriched pathways included those linked to wound healing, such as “Apoptosis-multiple species”, “Toll-like receptor signaling pathway”, and “C-type lectin receptor signaling pathway”. Additionally, the intersection targets were involved in various pathways related to inflammation, metabolic regulation, and cell growth, including “Lipid and atherosclerosis” and the “p53 signaling pathway”.

The combined results from GO and KEGG analyses suggest that B-CGT may promote diabetic wound healing through multiple synergistic pathways. For instance, apoptosis-related targets such as CASP8, CASP9, and CASP1 might exert effects by regulating cell apoptosis and the release of inflammatory mediators [42]. SIRT1 could potentially improve the wound microenvironment by modulating metabolism and fatty acid homeostasis [46]. Moreover, matrix metalloproteinases like MMP8 and MMP7 might facilitate tissue repair through the degradation and remodeling of the extracellular matrix. These findings provide a comprehensive understanding of the mechanisms underlying B-CGT’s role in diabetic wound healing.

#### 2.1.3. Integration and Analysis of the Drug-Target-Function-Pathway Network

Functional annotation and signaling pathway enrichment analysis of the intersecting targets of B-CGT were performed. A drug-target-function-pathway network was constructed to reveal its multidimensional mechanism of action (Figure 2C). In the network, targets such as CASP8, CASP9, CASP1, SIRT1, and MMP8 exhibited high connectivity and centrality, suggesting that these targets may play a critical role in the regulation of diabetic wound healing by B-CGT.

Functional annotation analysis showed that the targets were significantly enriched in functions such as “hydrolase activity”, “metallopeptidase activity”, and “protein-specific binding”. These functions are directly related to key processes in diabetic wound healing. For instance, MMP7 and MMP8, as matrix metalloproteinases, regulate the degradation and remodeling of the extracellular matrix, thereby providing the necessary conditions for cell migration and tissue repair. Apoptosis-related targets such as CASP8, CASP9, and CASP1 optimize the wound microenvironment by regulating cell apoptosis and the release of inflammatory factors.

Pathway enrichment analysis further indicated that B-CGT may exert its therapeutic effects through the synergistic action of multiple signaling pathways. The “Apoptosis—multiple species” pathway regulates cell apoptosis through CASP8 and CASP9, alleviating chronic inflammation in the wound healing process [47]. The “Toll-like receptor signaling pathway” modulates the inflammatory response and immune regulation through CASP8 and CTSK [48]. The “Regulation of lipolysis in adipocytes” and “Lipid and atherosclerosis” pathways improve metabolic homeostasis and optimize the wound microenvironment in diabetic patients through the action of SIRT1 and ADORA1 [49,50]. Additionally, the p53 signaling pathway enhances the proliferative and differentiative capacity of wound tissue by regulating the cell cycle and stress response. The C-type lectin receptor signaling pathway may enhance immune clearance through CASP8 and CASP1, promoting the healing process [51,52].

In summary, it can be clearly inferred that B-CGT achieves its effect in diabetic wound healing through the synergistic regulation of multiple targets and pathways. Its core mechanisms include the regulation of cell apoptosis to alleviate chronic inflammation, the promotion of tissue repair through extracellular matrix remodeling, and the optimization of the healing microenvironment through metabolic homeostasis.

### 2.2. Molecular Docking Evaluation

Molecular docking results indicate that B-CGT exhibits high binding affinity with key targets (Table 5). In particular, the targets SIRT1, CASP9, PTGS1, and MMP8 show significant Total Scores of 10.0932, 7.8708, 7.8977, and 7.9809, respectively. The high score for SIRT1 suggests that it may be a primary target of B-CGT. The binding site analysis reveals the formation of multiple hydrogen bonds and hydrophobic interactions, which enhance binding stability. Additionally, the high binding scores for CASP9 and PTGS1 suggest their potential roles in apoptosis regulation and inflammation pathways.

**Figure 3:**
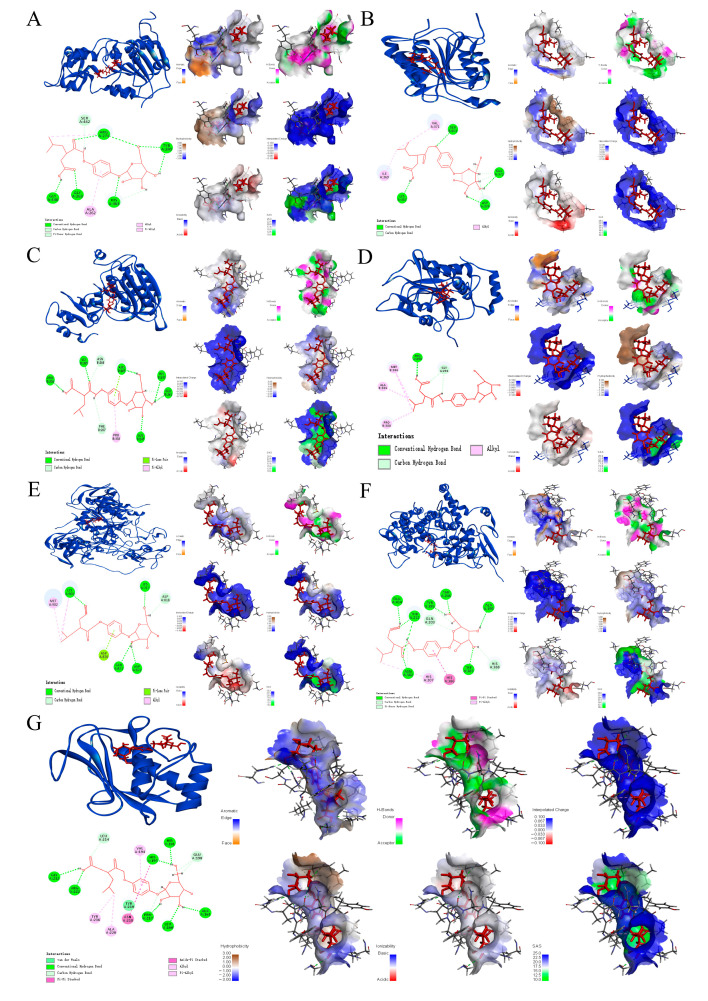
Molecular docking patterns of B-CGT active components and core targets. (**A**): Molecular docking pattern of SIRT1 with 2-isobutylmalic acid glucosoxybenzyl ester. (**B**): Molecular docking pattern of CASP8 with 2-isobutylmalic acid glucosoxybenzyl ester. (**C**): Molecular docking pattern of CASP9 with 2-isobutylmalic acid glucosoxybenzyl ester. (**D**): Molecular docking pattern of CASP1 with 2-isobutylmalic acid glucosoxybenzyl ester. (**E**): Molecular docking pattern of DNMT1 with 2-isobutylmalic acid glucosoxybenzyl ester. (**F**): Molecular docking pattern of PTGS1 with 2-isobutylmalic acid glucosoxybenzyl ester. (**G**): Molecular docking pattern of MMP8 with 2-isobutylmalic acid glucosoxybenzyl ester.

Molecular interaction analysis at the binding sites further elucidates the specific interaction patterns between B-CGT and the targets (Figure 3). For example, in the case of SIRT1, B-CGT stabilizes the binding through multiple hydrogen bonds with GLY440 and GLY263, as well as hydrophobic interactions (π-π stacking) with ARG274. Similarly, CASP9 enhances binding through several polar interactions and hydrophobic forces. MMP8 interacts with B-CGT through its metal-dependent active site, potentially playing a key role in matrix remodeling.

Crash value analysis reveals that the binding conformations for all targets exhibit low spatial conflicts (crash values range from −2.6568 to −1.3801), suggesting that the binding conformations are reasonable and stable. Polar value analysis also shows significant polar interactions in the binding with all targets (Polar values range from 3.9987 to 5.8399), further supporting the hypothesis that B-CGT regulates target activity through hydrogen bond networks and polar interactions.

Overall, the molecular docking results suggest that SIRT1, CASP9, PTGS1, and MMP8 are key targets through which B-CGT exerts its effects, involving biological processes such as cell apoptosis, inflammation regulation, and matrix remodeling. These findings provide direct evidence for the multi-target mechanism of B-CGT in diabetic wound healing and offer theoretical support for the development of B-CGT-based therapeutic strategies.

### 2.3. Antibacterial Effect

**Figure 4:**
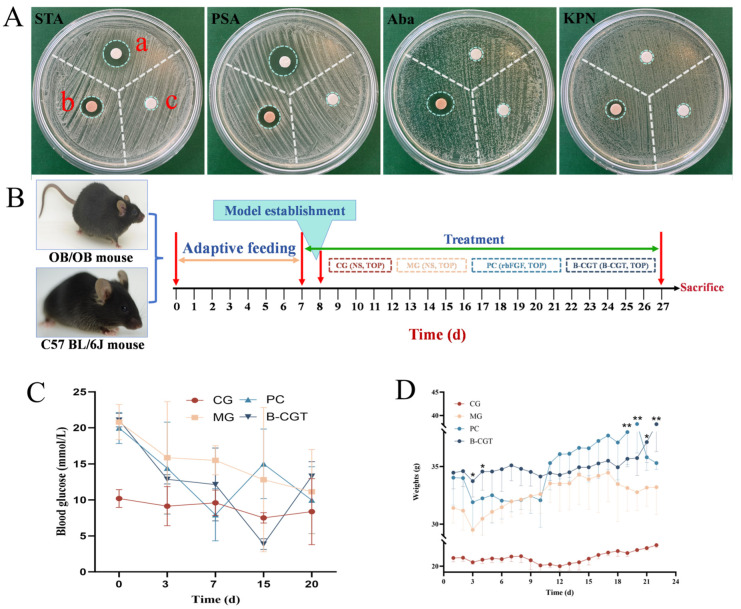
Antibacterial and mouse experimentation. (**A**): Antibacterial test results of B-CGT hydrogel (a represents amoxicillin, b represents B-CGT hydrogel extract, c represents the saline control). (**B**): Experimental procedure timeline for wound healing in diabetic mice. (**C**): Blood glucose fluctuations in mice across different time points during the experimental period. Although blood glucose levels fluctuated within each group, no statistically significant differences were observed overall. (**D**): Statistical analysis of body weight changes in mice across different time points. Significant differences were observed between the PC and B-CGT groups in the later stages of the experiment compared to the other groups, * *p* < 0.05, ** *p* < 0.01 vs. MG group.

As shown in Figure 4A, the B-CGT hydrogel extract exhibited significant antibacterial activity against four pathogenic bacteria (Staphylococcus aureus, Acinetobacter baumannii, Klebsiella pneumoniae, and Pseudomonas aeruginosa), outperforming the positive control, amoxicillin. Staphylococcus aureus is a common cause of skin and soft tissue infections, while Pseudomonas aeruginosa is frequently associated with burn wound infections [53,54]. Acinetobacter baumannii and Klebsiella pneumoniae, due to their multidrug resistance, have become recalcitrant pathogens in clinical treatments [55,56,57,58]. Although amoxicillin effectively inhibits Staphylococcus aureus and Pseudomonas aeruginosa, it is ineffective against Acinetobacter baumannii and Klebsiella pneumoniae. In contrast, the B-CGT hydrogel extract demonstrated robust antibacterial activity against all experimental strains, indicating its broad-spectrum antibacterial potential. This suggests that it could effectively prevent wound infections, particularly when faced with resistant bacteria.

### 2.4. Effect of B-CGT Hydrogel on Body Weight and Blood Glucose in ob/ob Mice

The timeline for the diabetic mouse wound healing experiment is shown in Figure 4B. The ob/ob mouse model exhibits characteristics similar to those of adult diabetic patients and is widely used to evaluate the link between diabetes and impaired wound healing [59]. In this experiment, ob/ob mice were used to investigate the effects of B-CGT hydrogel on diabetic wound healing. Figure 4C,D show the changes in body weight and blood glucose levels in different groups of mice after modeling. Post-modeling, the body weight of ob/ob mice was approximately 1.5 times that of C57BL/6J wild-type mice. Following treatment, body weight in the MG, PC, and B-CGT hydrogel groups increased steadily. After day 18, mice in the PC and B-CGT groups exhibited significantly higher body weights compared to the MG (*p* < 0.05), indicating improved physiological status due to the treatment. The C57BL/6J control group showed slower weight gain, likely due to their normal growth characteristics. Although blood glucose levels fluctuated in the MG, PC, and B-CGT groups at different time points, no significant differences were observed compared to the MG.

### 2.5. Wound Healing in ob/ob Mice

**Figure 5:**
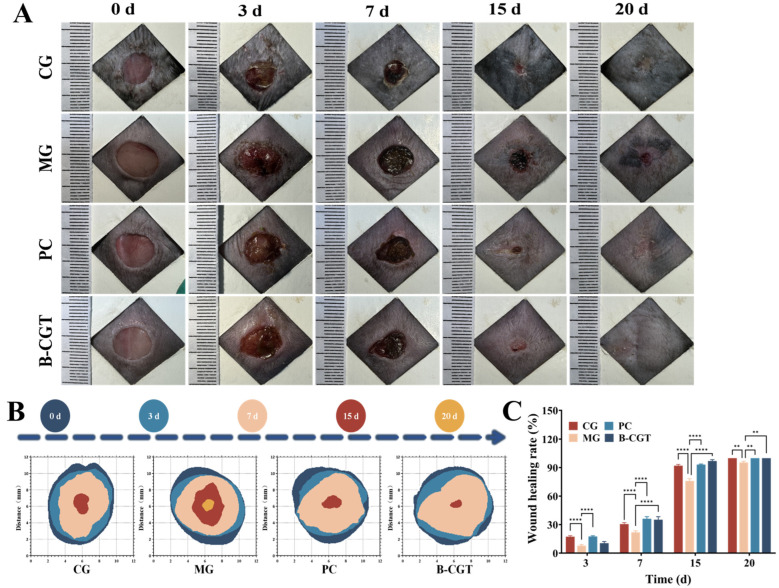
Wound changes in experimental mice during the study period. (**A**): Representative images of wound healing in experimental mice from day 0, 3, 7, 15, and 20 in each group. (**B**): A schematic of wound area changes over time for each group of mice on days 0, 3, 7, 15, and 20. (**C**): Quantitative data of relative wound area for each group of mice on days 3, 7, 15, and 20 (*n* = 3), ** *p* < 0.01, **** *p* < 0.0001.

Delayed wound healing in diabetes is caused by multiple factors, including the persistent high expression of inflammatory factors and the lack of growth factors, which result in prolonged chronic inflammation at the wound site, hindering the repair process [60]. Imbalance of immune homeostasis in diabetic wounds leads to continuous inflammation, impaired angiogenesis, and reduced epithelial repair capacity, thereby significantly delaying the healing process [61]. To evaluate the promoting effect of B-CGT hydrogel on diabetic wound healing, a full-thickness skin wound model was used. Figure 5A illustrates the entire healing process of ob/ob diabetic mice from wound excision to healing. Figure 5B presents a time-lapse chart of wound area changes in different groups. The results indicated that the wound healing rates in the PC and B-CGT hydrogel groups were significantly higher than those in the MG. On day 3 of treatment, wound closure rates in the CG, PC, and B-CGT hydrogel groups were 17.26 ± 1.12%, 17.57 ± 0.73%, and 10.50 ± 1.64%, respectively, significantly higher than the MG (7.88 ± 1.20%) (*p* < 0.05). The MG, without any treatment, exhibited a significantly slower wound-healing rate due to immune dysfunction, resulting in prolonged inflammation at the wound site.

Compared to the CG, the wound healing rates in the PC and B-CGT hydrogel groups were significantly accelerated. Notably, the treatment effects in the PC and B-CGT groups were very similar, both effectively promoting wound healing in diabetic mice. As the treatment progressed, the wound healing effect in the B-CGT hydrogel group became the most pronounced. On day 15, the wound healing rate in the B-CGT group exceeded 95% (96.89 ± 1.47%), outperforming the PC group (93.18 ± 0.61%) and the CG (92.05 ± 1.26%). In contrast, the MG had a wound healing rate of only 76.04 ± 2.30%, significantly lagging behind the other groups. On day 20, the wounds in the B-CGT, PC, and CGs were almost completely healed, while the MG achieved a healing rate of only 95.36 ± 1.31%. These results suggest that B-CGT hydrogel significantly promotes the healing of diabetic wounds and accelerates the healing process.

### 2.6. Granulation Tissue Repair and Epidermal Growth

**Figure 6:**
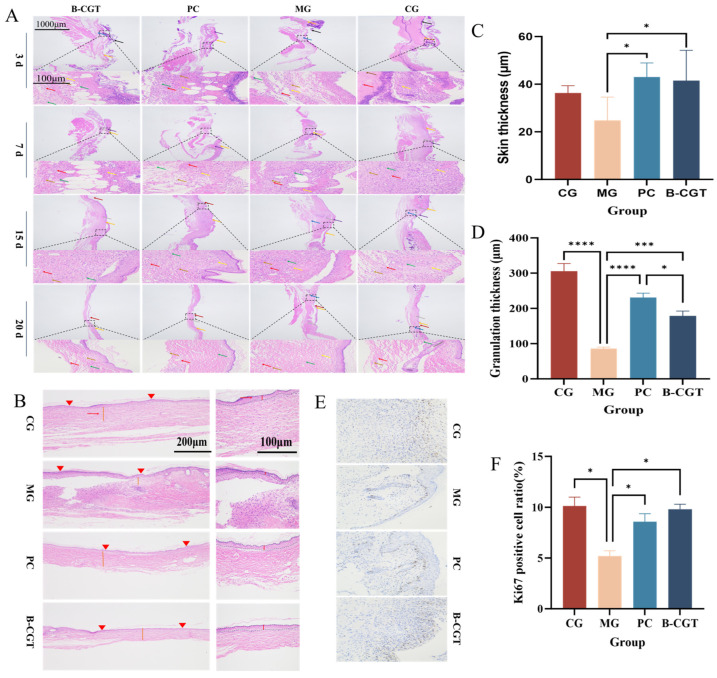
Granulation tissue repair and epidermal growth. (**A**): Representative HE-stained images of wound tissue at different time points in each experimental group (red arrow, yellow arrow, green arrow, blue arrow, brown arrow, purple arrow, black arrow). (**B**): Schematic of the measurement of epidermal thickness and granulation tissue thickness in wound tissue after HE staining in each experimental group. (**C**): Statistical analysis of epidermal thickness in wound tissue of each experimental group on day 20 (*n* = 3). The line indicates the thickness of the epidermis, and the arrow indicates the granulation tissue. (**D**): Statistical analysis of granulation tissue thickness in wound tissue of each experimental group on day 20 (*n* = 3). (**E**): Ki67 immunohistochemical images of wound tissue from each experimental group on day 7. (**F**): Quantitative Ki67 immunohistochemical results of wound tissue from each experimental group on day 7. * *p* < 0.05, *** *p* < 0.001, **** *p* < 0.0001.

Granulation tissue plays a crucial role in the healing process of wounds, particularly in the presence of inflammatory responses [62,63]. With the onset of inflammation, fibroblast proliferation promotes the formation of granulation tissue, which provides a growth matrix and supports the generation of new blood vessels [64,65]. Therefore, the thickness of granulation tissue is often used as a key indicator to evaluate the effectiveness of wound repair. As shown in Figure 6A, by day 20 of treatment, the wounds in all groups were nearly healed. HE staining analysis revealed that the granulation tissue thickness in the MG was significantly lower compared to the other groups. In contrast, the granulation tissue in the B-CGT hydrogel group was markedly thicker (*p* < 0.001), suggesting that B-CGT hydrogel effectively promotes wound healing (Figure 6D).

During the wound healing process, the dermis provides structural support, while the formation of granulation tissue offers growth factors and an extracellular matrix (ECM) to the wound site. Additionally, the epidermis rapidly covers the wound, forming an early functional barrier to prevent moisture loss and reduce the risk of infection. The formation and maturation of granulation tissue directly influence epidermal regeneration, while the reconstruction of the dermis lays the foundation for the health and functional recovery of the epidermis [66]. Therefore, epidermal regeneration typically precedes dermal repair and is considered a critical step in skin wound healing. To further assess the epithelialization process and granulation tissue formation, we observed wound healing in the different groups on day 20 post-treatment. Figure 6B presents the measurement results for epidermal and granulation tissue thickness. Compared to the MG, the CG, PC, and B-CGT hydrogel groups exhibited relatively intact epidermises, while the MG still displayed open wounds after 7 days of treatment. On day 20, the epithelial formation in the B-CGT hydrogel group was significantly superior to that in the MG (*p* < 0.05) (Figure 5C). During wound healing, epidermal cells must proliferate rapidly to cover the wound, and Ki67, a marker of cell proliferation, can reflect cellular proliferation and regeneration during the repair process. Figure 6E,F show that, compared to the MG, the B-CGT hydrogel treatment group had significantly higher Ki67 expression (*p* < 0.05). This further confirms that B-CGT hydrogel effectively promotes epidermal growth, accelerating wound healing.

### 2.7. In Vivo Inflammation and Macrophage Expression

**Figure 7:**
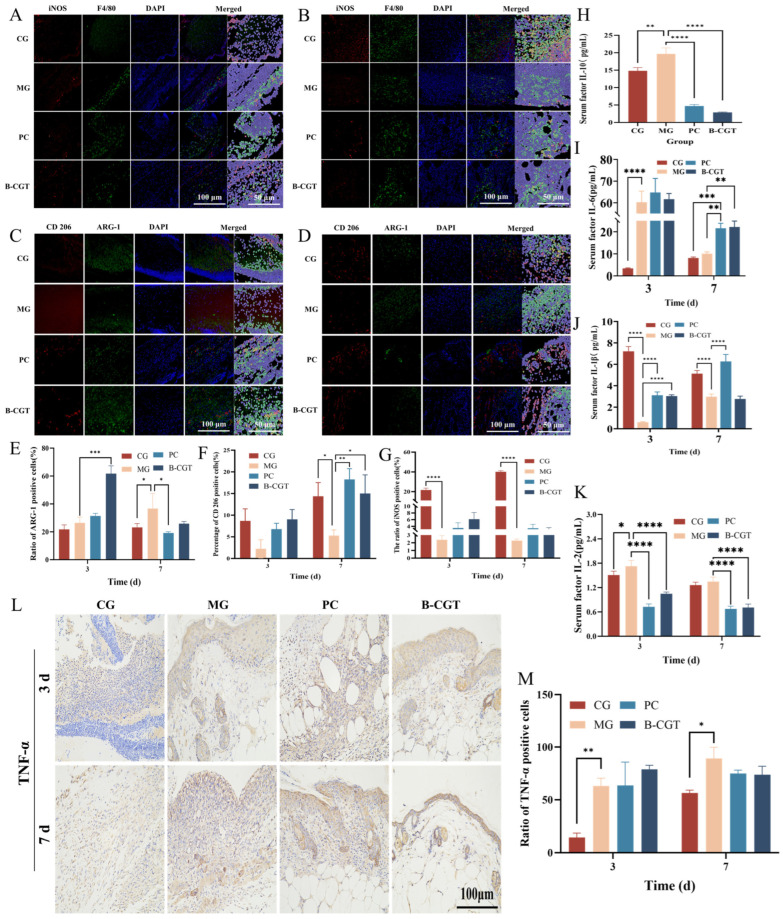
Changes in in vivo inflammation and macrophage expression in treatment groups. (**A**): Immunofluorescence staining for iNOS + F4/80 in wound tissue of each group on day 3 of treatment. (**B**): Immunofluorescence staining for iNOS + F4/80 in wound tissue of each group on day 7 of treatment. (**C**): Immunofluorescence staining for CD206 + ARG-1 in wound tissue of each group on day 3 of treatment. (**D**): Immunofluorescence staining for CD206 + ARG-1 in wound tissue of each group on day 7 of treatment. (**E**): Quantitative analysis of ARG-1-positive cell ratios on days 3 and 7 of treatment (*n* = 3). (**F**): Quantitative analysis of CD206-positive cell ratios on days 3 and 7 of treatment (*n* = 3). (**G**): Quantitative analysis of iNOS-positive cell ratios on days 3 and 7 of treatment (*n* = 3). (**H**): Quantitative analysis of serum IL-10 levels in each group on day 7 (*n* = 3). (**I**): Quantitative analysis of serum IL-6 levels in each group on days 3 and 7 (*n* = 3). (**J**): Quantitative analysis of serum IL-1β levels in each group on days 3 and 7 (*n* = 3). (**K**): Quantitative analysis of serum IL-2 levels in each group on days 3 and 7 (*n* = 3). (**L**): Immunohistochemical staining for TNF-α in wound tissue on days 3 and 7 of treatment. (**M**): Quantitative analysis of TNF-α expression in wound tissue on days 3 and 7 of treatment (*n* = 3). * *p* < 0.05, ** *p* < 0.01, *** *p* < 0.001, **** *p* < 0.0001.

The initiation of the inflammatory response is the first step in wound healing and directly influences subsequent regenerative stages, which are critical for the final healing outcome [26,67]. In this study, F4/80 was used as a macrophage marker, while iNOS, CD206, and ARG-1 served as indicators for M1 and M2 macrophages, respectively, to assess changes in the inflammatory response in diabetic wounds after treatment. Figure 7A–D show the immunofluorescence staining results for iNOS, CD206, and ARG-1 expression on the wound surface on days 3 and 7 post-surgery. The results indicated that on day 3, the M2 macrophage markers (CD206 or ARG-1) in the B-CGT hydrogel treatment group were expressed at significantly higher levels compared to the other groups (Figure 7E,F). On both days 3 and 7, the MG exhibited significantly lower expression of iNOS and CD206 compared to the CG, PC, and B-CGT groups. Notably, on day 7, ARG-1 expression was significantly upregulated, indicating that the model group failed to clear necrotic tissue from the wound during the inflammatory phase, hindering the normal transition to the healing stage (Figure 7G). Compared to the CG, the B-CGT group showed a significant reduction in iNOS expression (*p* < 0.001). Although CD206 and ARG-1 upregulation was evident, it did not reach statistical significance (*p* > 0.05).

To further confirm the role of B-CGT hydrogel in promoting the transition to an effective healing phase, we assessed the levels of chemokines in mouse serum using enzyme-linked immunosorbent assay (ELISA). Pro-inflammatory cytokines play a crucial role in the initiation and progression of the inflammatory response, while the anti-inflammatory cytokine IL-10 can inhibit the synthesis and release of various pro-inflammatory factors, thereby effectively reducing the intensity of inflammation [68,69]. Figure 7H shows that the serum IL-10 concentration in the B-CGT group was significantly lower than that in the MG (*p* < 0.0001), while the levels of pro-inflammatory cytokines IL-1β and IL-6 were also significantly lower in the B-CGT group compared to the MG (Figure 7I,J). Additionally, IL-2, a key factor promoting T cell proliferation and differentiation, was expressed at lower levels in the B-CGT and CGs compared to the MG, with significant differences observed (Figure 7K). Figure 7L presents the immunohistochemical staining results for TNF-α expression on the wound surface. On day 3, the TNF-α-positive cell proportion was lowest in the normal group (*p* < 0.01). By day 7, the model group exhibited significantly higher TNF-α expression compared to the normal group (*p* < 0.05). It was slightly higher than the positive control and B-CGT groups, although the difference was not statistically significant (*p* > 0.05). These results suggest that both B-CGT hydrogel and the positive drug treatment significantly reduce TNF-α expression, thereby promoting wound healing (Figure 7M).

### 2.8. In Vivo Collagen Deposition and Neovascularization

Fibroblasts are the predominant cell type in granulation tissue. Under the stimulation of TGF-β, fibroblasts undergo structural and functional changes, differentiating into myofibroblasts that synthesize collagen and other extracellular matrix (ECM) components. This process plays a central role in collagen synthesis and tissue repair [70,71,72]. Moreover, collagen is a major component of the ECM, and mature blood vessels provide essential metabolic support to cells, promoting further collagen synthesis [73,74]. Collagen deposition and neovascularization are critical for ECM remodeling and functional recovery [75].

**Figure 8:**
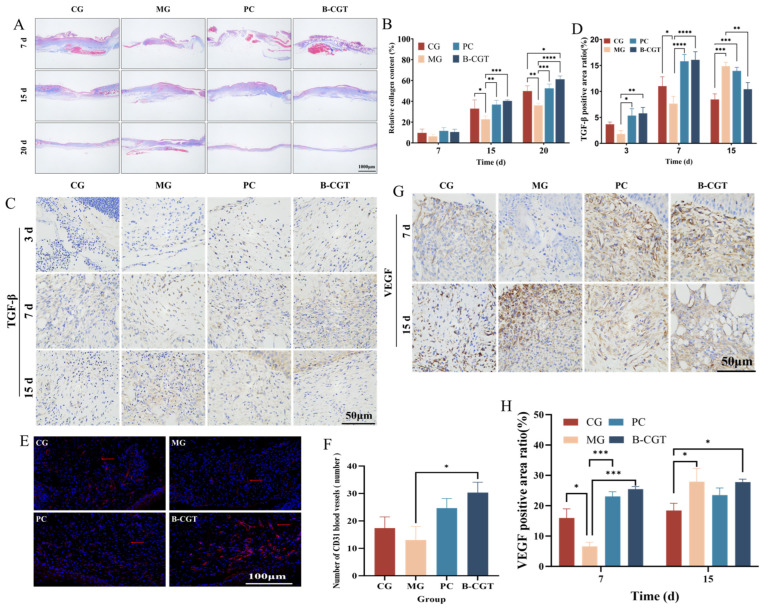
Collagen deposition and neovascularization in different treatment groups. (**A**): Masson’s trichrome staining of wound tissue from each experimental group on days 7, 15, and 20 of treatment. (**B**): Quantitative analysis of collagen fiber density in wound tissue from each experimental group on days 7, 15, and 20 (*n* = 3). (**C**): TGF-β immunohistochemical staining in wound tissue from each experimental group on days 3, 7, and 15. (**D**): Quantitative analysis of TGF-β-positive areas in wound tissue from each experimental group on days 3, 7, and 15 (*n* = 3). (**E**): CD31 immunofluorescence staining of wound tissue from each experimental group on day 7. (**F**): Quantitative analysis of new blood vessel numbers (MVD) in wound tissue from each experimental group on day 7 (*n* = 3). (**G**): VEGF immunohistochemical staining in wound tissue from each experimental group on days 7 and 15. (**H**): Quantitative analysis of VEGF positive area ratios in wound tissue from each experimental group on days 7 and 15 (*n* = 3). * *p* < 0.05, ** *p* < 0.01, *** *p* < 0.001, **** *p* < 0.0001.

Figure 8A,B shows that, by day 7 of treatment, the CG exhibited higher fiber density, followed by the PC and B-CGT hydrogel groups. In contrast, the MG showed lower collagen deposition due to prolonged hyperglycemia and a high-fat environment. After 15 days of treatment, the collagen fiber density in the B-CGT hydrogel group surpassed that in the CG and was significantly higher than in the MG (*p* < 0.001). On day 20, the collagen density in the B-CGT hydrogel group was significantly higher than in the other groups (*p* < 0.05), indicating that B-CGT hydrogel significantly promotes collagen deposition and accelerates wound healing.

TGF-β is an important growth factor that stimulates fibroblast migration and proliferation at the injury site. It also has chemotactic effects on monocytes and supports angiogenesis [76]. To further evaluate the effect of B-CGT hydrogel on wound healing, we assessed TGF-β expression in wound tissue through immunohistochemical staining (Figure 8C). The results showed that on days 3 and 7 of treatment, the B-CGT hydrogel group had significantly higher TGF-β-positive areas compared to the MG. By day 15, TGF-β expression in the CG, PC, and B-CGT groups gradually decreased, while the TGF-β levels in the MG continued to rise. This may be due to the sustained inflammatory state in the MG’s wounds, obstructing the expression of transforming growth factors. Figure 8D further confirmed this result: on day 3, the B-CGT hydrogel group exhibited significantly larger TGF-β-positive areas compared to the MG (*p* < 0.01). On day 7, the positive area ratios in the PC and B-CGT groups were significantly higher than in the MG (*p* < 0.0001). After day 15 of treatment, TGF-β expression generally decreased in all groups, but in the MG, it continued to rise, suggesting that its wound had not progressed to the healing phase. The B-CGT hydrogel group showed significantly lower TGF-β expression compared to the MG (*p* < 0.01), further proving the role of B-CGT hydrogel in regulating TGF-β expression and promoting wound repair.

To evaluate angiogenesis, we assessed the neovascularization in diabetic wounds using CD31 immunofluorescence staining on day 7 of treatment (Figure 8E). The results showed that, compared to the MG, CD31 expression was significantly increased in the B-CGT hydrogel and positive drug groups, indicating the formation of more mature new blood vessels in these two groups. Figure 8F shows that the number of new blood vessels (MVD) in the B-CGT hydrogel group was significantly higher than in the MG (*p* < 0.05). Vascular endothelial cells migrate to the wound site and connect with each other under the induction of VEGF, ultimately forming mature blood vessels (Figure 8G). On day 7 of treatment, VEGF expression in the B-CGT hydrogel group was significantly higher than in the MG, with a marked difference (*p* < 0.001) (Figure 8H), indicating that B-CGT hydrogel effectively promotes angiogenesis.

### 2.9. Cytoplasmic Matrix Remodeling

Collagen is a key structural component of the ECM [77,78]. Collagen I plays a central role in the skin by providing tensile strength and support, while Collagen III is critical for skin tissue repair due to its flexibility and elasticity [79,80,81]. Collagen typically exists in thick, bundled fibers. As collagen deposits increase, the wound scar gradually matures, and structural remodeling occurs during the process of fibroblast conversion into myofibroblasts [82,83]. The synthesis and remodeling of the ECM occur synchronously with granulation tissue formation, and this process is prolonged [84]. As newly synthesized Collagen III is arranged, existing collagen is degraded by collagenases and replaced by Collagen I, which provides greater mechanical strength and enhances skin tension. As Collagen III increases and transitions to Collagen I, collagen fibers reorganize into larger bundles, replacing the old ECM [85,86,87,88]. During this process, the growth factor TGF-β promotes fibroblast migration to the injury site and induces their differentiation into myofibroblasts, thus driving ECM remodeling and wound contraction [89,90,91].

**Figure 9:**
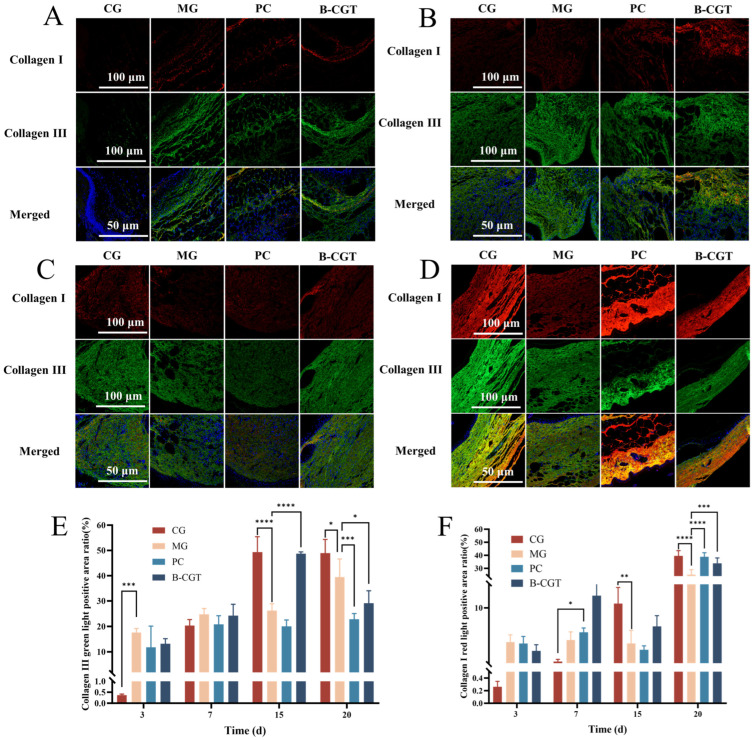
Cytoplasmic matrix remodeling in different treatment groups. (**A**): Dual immunofluorescence staining of Collagen I and Collagen III in wound tissue from each experimental group on day 3 of treatment. (**B**): Dual immunofluorescence staining of Collagen I and Collagen III in wound tissue from each experimental group on day 7 of treatment. (**C**): Dual immunofluorescence staining of Collagen I and Collagen III in wound tissue from each experimental group on day 15 of treatment. (**D**): Dual immunofluorescence staining of Collagen I and Collagen III in wound tissue from each experimental group on day 20 of treatment. (**E**): Quantitative analysis of the positive area ratio of Collagen III in wound tissue from each experimental group on days 3, 7, 15, and 20. (**F**): Quantitative analysis of the positive area ratio of Collagen I in wound tissue from each experimental group on days 3, 7, 15, and 20. * *p* < 0.05, ** *p* < 0.01, *** *p* < 0.001, **** *p* < 0.0001.

On days 3, 7, 15, and 20 post-treatment, we evaluated the production of Collagen I and Collagen III in diabetic wounds using dual immunofluorescence staining (Figure 9A–D). The results showed that on day 3, Collagen I expression was low, while Collagen III expression was higher and overall greater than in the CG. By day 7, Collagen I staining in the B-CGT hydrogel group was the most intense, while Collagen III expression was slightly stronger in the MG and B-CGT groups. After 15 days of treatment, Collagen III expression in the B-CGT hydrogel group was the strongest, followed by the CG. The PC and MGs showed lower and slower increases in expression. In contrast, Collagen I staining was more pronounced in the CG, while the B-CGT group showed the strongest Collagen I expression, possibly due to the impaired immune function in diabetic mice. These results indicate that B-CGT hydrogel effectively promotes Collagen I synthesis.

By day 20, the wound entered the reconstruction phase, and a significant enhancement in Collagen I staining was observed, while Collagen III staining decreased, likely due to the conversion of Collagen III to Collagen I. Notably, the PC group showed relatively stable Collagen I staining throughout the treatment period, with an increase in Collagen I expression during the reconstruction phase. Figure 9E,F demonstrates that the positive expression rates of Collagen I and Collagen III followed consistent trends, with the B-CGT hydrogel group significantly regulating the expression of both collagen types and promoting wound healing.

### 2.10. Degree of Oxidative Damage in the Wound

**Figure 10:**
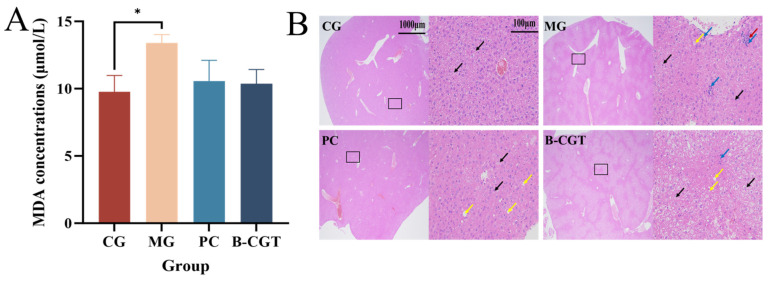
Oxidative damage in the wound and in vivo toxicity testing of B-CGT hydrogel. (**A**): Quantitative analysis of MDA levels in serum on day 7 post-treatment in each experimental group. * *p* < 0.05. (**B**): HE pathological analysis of liver tissue on day 20 post-treatment in each experimental group. Black arrows indicate cellular edema, red arrows point to vascular congestion, yellow arrows indicate rare fatty degeneration of hepatocytes, and blue arrows indicate lymphocytic infiltration.

Malondialdehyde (MDA) is a commonly used marker of oxidative damage and is widely employed to assess the body’s antioxidant capacity [92]. Higher MDA levels typically indicate increased reactive oxygen species (ROS) levels and a weakened antioxidant defense. To evaluate the antioxidant properties of B-CGT hydrogel, we measured the MDA levels in the serum of mice. The results showed that the MDA content in the CG was significantly lower than in the MG (*p* < 0.05). Furthermore, the MDA levels in the B-CGT and PC groups were lower than those in the MG, with a trend toward reduced MDA levels in the wound. However, no significant statistical differences were observed between the B-CGT and the model group (*p* > 0.05) (Figure 10A). These findings suggest that B-CGT hydrogel may promote wound healing by alleviating oxidative stress at the wound site.

### 2.11. In Vivo Toxicity of the Hydrogel

To further assess whether B-CGT hydrogel caused any adverse effects on the organs of mice during treatment, we examined the organ changes after hydrogel application. Given that diabetic mice may already have pre-existing organ damage, this could affect the evaluation of hydrogel toxicity on the organs. Figure 10B shows that on day 20 post-surgery, the liver tissue of CG mice exhibited typical normal architecture, with hepatocytes arranged orderly and intact cellular structures. No signs of cellular edema, vascular congestion, or fatty degeneration were observed, and there were no significant pathological changes or toxic reactions. Compared to the MG, no significant differences were observed in the pathological indicators of the PC and B-CGT hydrogel groups, and no liver damage was observed. These results indicate that B-CGT hydrogel did not cause apparent effects on the liver of experimental mice during the 20-day treatment, suggesting it has good safety.

## 3. Conclusions

The repair of chronic diabetic wounds is a complex process involving immune regulation, angiogenesis, and tissue remodeling. However, current treatments have limited efficacy in the multi-target regulation of these processes. This study systematically investigates the multi-dimensional mechanisms of B-CGT hydrogel in diabetic wound healing through network pharmacology, molecular docking, and in vivo experiments. The findings demonstrate the potential of B-CGT hydrogel as a next-generation therapeutic strategy.

Network pharmacology analysis revealed that the mechanism of B-CGT involves several core targets, such as SIRT1, CASP8, and MMP8. These targets are enriched in key signaling pathways related to apoptosis regulation, inflammatory response, and extracellular matrix remodeling. Molecular docking results validated the high-affinity binding of B-CGT to these targets, suggesting therapeutic effects through modulation of cell apoptosis, immune responses, and matrix remodeling.

In vivo experiments demonstrated that B-CGT hydrogel significantly accelerated wound healing in diabetic mice. This was characterized by faster wound closure, enhanced angiogenesis, improved collagen deposition, and increased granulation tissue formation. Additionally, B-CGT hydrogel modulated macrophage polarization, promoting the conversion of M1 macrophages to M2 macrophages, which effectively alleviated the chronic inflammatory state of diabetic wounds. The upregulation of VEGF and its antioxidant effects further optimized the wound microenvironment, enhancing the wound repair capacity.

This study integrates multi-level theoretical predictions with experimental validation, highlighting the unique advantages of B-CGT hydrogel in diabetic wound therapy. By addressing immune imbalance and insufficient angiogenesis in diabetic wounds through multi-target and multi-pathway cooperation, it demonstrates superior therapeutic effects and promising clinical potential. This study not only provides an efficient and safe novel strategy for the treatment of diabetic chronic wounds but also opens new avenues for the application of natural products in the development of multifunctional therapeutic materials. Future research will include clinical studies to further validate its efficacy and safety, contributing to improved quality of life for diabetic patients.

## 4. Materials and Methods

### 4.1. Network Pharmacology Study

#### 4.1.1. Prediction of Potential Targets of B-CGT

The chemical structure of B-CGT was retrieved from the PubChem database to obtain its standardized representation. Based on the molecular formula and structural information of B-CGT, the online tool TargetNet (http://targetnet.scbdd.com/calcnet/index/, accessed on 12 October 2024) was used to predict potential targets, with an AUC greater than or equal to 0.7 as the selection criterion [93]. The relationships between the selected targets and the drug were visualized using Cytoscape software, creating a target–drug network.

#### 4.1.2. Venn Analysis of Drug–Disease Intersection Targets and Core Target Selection

Targets associated with diabetic wound healing were retrieved from the GeneCards database (http://www.genecards.org, accessed on 12 October 2024) by searching with the keyword “diabetic wound healing”. Targets with a score greater than 0 were selected, as these are strongly related to the disease and may represent key driving factors or have been widely reported in the literature. The relationships between the selected targets and the disease were visualized using Cytoscape, forming a target–disease network.

A list of drug targets and disease-related targets was compiled and input into the online tool Evenn (http://www.ehbio.com/test/venn/#/, accessed on 12 October 2024), to conduct Venn analysis for the drug–disease intersection targets. PPI (protein–protein interaction) information for the drug–disease targets was obtained using the CytoNCA plugin in Cytoscape, and their shared targets were identified. Core targets were selected based on the following criteria: degree centrality (DC), betweenness centrality (BC), closeness centrality (CC), eigenvector centrality (EC), network centrality (NC), and local average connectivity (LAC), all of which must be greater than the median value.

#### 4.1.3. Functional Annotation and Enrichment Analysis

To further explore the potential mechanisms of B-CGT, functional annotation and pathway enrichment analysis were conducted on its intersecting targets. Gene Ontology (GO) analysis of the intersecting targets was performed using the GDI Bioinformatics platform (https://www.genedenovo.com/, accessed on 13 October 2024), focusing on biological processes (BP), cellular components (CC), and molecular functions (MF). Key functions related to diabetic wound healing were identified. Additionally, pathway enrichment analysis was carried out on the intersecting targets using the same platform to identify significantly enriched signaling pathways. Particular attention was given to pathways related to diabetic wound healing, and the potential pathways through which B-CGT might exert its effects in the wound healing process of diabetic mice were analyzed.

#### 4.1.4. Integration and Analysis of the Drug-Target-Function-Pathway Network

Based on the enrichment analysis of the targets’ functions and signaling pathways, the functional information of the targets was integrated with the associated pathways to construct a drug-target-function-pathway network. This network was visualized using Cytoscape software, which illustrates the complex relationships between the targets, functions, and pathways, thereby elucidating the potential mechanisms of action of B-CGT. Through the analysis of this network, the underlying molecular mechanisms of B-CGT in diabetic wound healing were explored, providing a systematic theoretical basis for its multi-target, multi-functional, and multi-pathway action model.

### 4.2. Molecular Docking

To explore the interactions between the B-CGT molecule and key targets, molecular docking was performed using SYBYL-X 2.0 software, and the results were visualized using Discovery Studio 2019. The three-dimensional structure of the receptor protein was obtained from the PDB database and preprocessed, which involved the removal of water molecules and the addition of hydrogen atoms. The ligand molecule was optimized using the MMFF94 force field to obtain the lowest energy conformation. Molecular docking simulations were conducted using the “Flexible Docking” mode of the Surflex-Dock module. The optimal binding conformation was selected based on the Total Score scoring function. Intermolecular interactions, such as hydrogen bonding and hydrophobic interactions at the binding site, were visualized using Discovery Studio, providing theoretical support for the study of the drug’s mechanism of action.

### 4.3. Antibacterial Test

In this experiment, four common pathogenic bacteria were selected: *Staphylococcus aureus*, *Acinetobacter baumannii*, *Klebsiella pneumoniae*, and *Pseudomonas aeruginosa*. All bacterial strains were provided by Guizhou University of Traditional Chinese Medicine. The antibacterial activity of B-CGT hydrogel extract was evaluated using the agar diffusion method (disk diffusion test). Specifically, 50 µL of standardized bacterial suspension (1 × 10^8^ CFU/mL) was evenly spread on the surface of MH agar plates (Guangzhou Huankai Microbial Technology Co., Ltd., Guangzhou, China batch number: 230919A20) using a sterile cotton swab. Then, antibiotic paper disks treated with different solutions were placed on the agar surface. The plates were incubated at 37 °C for 24 h in a constant-temperature incubator (DHP-9256, Shandong Aolai Bo Instrument Co., Ltd., Jinan, China). After incubation, the inhibition zone diameter (mm) was measured using a vernier caliper to assess antibacterial efficacy. Experimental results were presented as the mean of three independent replicates.

### 4.4. In Vivo Wound Healing Experiment

#### 4.4.1. Animals

The ob/ob mice, a commonly used obesity model for studying type 2 diabetes, exhibit characteristics similar to adult-onset diabetes. These mice are homozygous and share the same genetic background as the wild-type C57BL/6J mice. All experimental male mice (6 weeks old) were purchased from Beijing Huafukang Biotechnology Co., Ltd. (Beijing, China). Mice were housed in SPF-grade animal facilities with a controlled temperature of 22 °C and a 12 h light/dark cycle. Each mouse was individually housed and provided with ad libitum access to food and water. To allow for acclimatization, the mice were given a 1-week adaptation period before the experiment. All procedures and experiments were approved by the Animal Ethics Committee of Guizhou University of Traditional Chinese Medicine (Animal Experiment Ethics Approval No. 2024057).

#### 4.4.2. Full-Thickness Skin Wound Model

The day before surgery, the hair on the dorsal side of the mice was shaved, and the area was disinfected. On the following day, anesthesia was induced using isoflurane (batch number: 2024012501, Shandong Antemuyu Technology Co., Ltd., Jinan, China), and the surgical area was disinfected. A full-thickness skin wound of approximately 10 mm in diameter was created on the back of each mouse. After the surgery, the wound was washed with physiological saline, and the mice were individually housed. The body weight and blood glucose levels of the mice were regularly monitored.

#### 4.4.3. Animal Grouping and Treatment

The mice were randomly divided into four groups: the wild-type C57BL/6J mice as the control group (CG), ob/ob diabetic mice as the model group (MG), Betafusin (also known as bovine basic fibroblast growth factor gel, batch number: S20040001, Zhuhai Yisheng Biopharmaceutical Co., Ltd., Zhuhai, China) as the positive control group (PC), and the B-CGT hydrogel group. During the treatment, the drugs or control solutions were applied once daily. On the first and second days post-surgery, a thick layer of the drug was applied to the wound area to absorb the exudate during the inflammatory phase of wound healing. Subsequently, the wound was covered daily. The model and control groups were treated with physiological saline. Wound photographs were taken on days 0, 3, 7, 15, and 20 post-surgery. The wound area and edge perimeter were quantified using ImageJ 10.0 software, and the percentage of the non-healed area was calculated using the following formula:

(1) $$S\%=\frac{A_{t}}{A_{0}}\times 100\%$$

where A_0_ and A_t_ represent the non-healed areas on day 3 and on days 7, 15, and 20, respectively.

#### 4.4.4. Histological Analysis by Hematoxylin and Eosin (HE) Staining

On days 3, 7, 15, and 20 post-treatment, tissue samples from the wound area were collected for histological evaluation of wound healing by HE staining. First, regenerated dorsal skin tissues were sectioned, fixed in 4% paraformaldehyde, and embedded in paraffin for subsequent staining analysis. Some samples were stored frozen at −80 °C. HE staining was performed using a Hematoxylin and Eosin Staining Kit (batch number: G1076, Wuhan Seville Biotech Co., Ltd., Wuhan, China) following the manufacturer’s instructions. After deparaffinization, the sections were dehydrated through graded ethanol (100%, 95%, and 75%). The sections were then immersed in hematoxylin solution for 5 min, followed by rinsing with distilled water and eosin staining for 15 s. After dehydration in anhydrous ethanol and clearing in xylene, the sections were mounted with neutral gum. Stained sections were observed under a Nikon ECLIPSE E100 optical microscope (Nikon Corporation, Tokyo, Japan), and granulation tissue and epidermal thickness were quantified.

#### 4.4.5. Masson’s Trichrome Staining Analysis

The deparaffinization and rehydration procedures for Masson’s trichrome staining were identical to those for HE staining. Masson’s trichrome staining was performed using a Masson’s Tricolor Staining Kit (batch number: G1006, Wuhan Seville Biotech Co., Ltd.) according to the manufacturer’s protocol to assess the presence of fibroblasts in the tissue. Sections were first soaked in a 2% potassium dichromate solution overnight for mordanting. The sections were then washed with distilled water and stained in Weigert’s iron hematoxylin solution (a mixture of equal volumes of Masson’s B and C solutions) for 1 min. After washing with distilled water, the sections were differentiated in differentiation solution for 5 s and stained with acid fuchsin for 6 min. The sections were then immersed in 1% phosphomolybdic acid for 1 min, followed by staining with 2% aniline blue solution for 30 s. After differentiation with 1% acetic acid and dehydration in anhydrous ethanol, the sections were mounted. Sections were observed and photographed under a Nikon ECLIPSE E100 optical microscope. Quantitative analysis was performed using ImageJ software.

#### 4.4.6. Immunofluorescence Analysis

For in vivo immunofluorescence analysis, skin tissue sections were first deparaffinized, rehydrated, and subjected to antigen retrieval. The sections were then treated with 3% hydrogen peroxide solution at room temperature, protected from light for 25 min to eliminate endogenous peroxidase activity. Following this, the sections were washed three times with PBS and blocked with 3% BSA at room temperature for 30 min to reduce non-specific binding. Next, the sections were incubated overnight at 4 °C with primary antibodies against Collagen I (1:4000, batch number: GB 11022-3, Wuhan Seville Biotech Co., Ltd., Wuhan, China), Collagen III (1:5000, batch number: GB 111629, Servicebio, Wuhan, China), CD 206 (1:2000, batch number: GB 113497, Servicebio), iNOS (1:1000, batch number: GB 15323, Servicebio), ARG-1 (1:5000, batch number: GB 11285, Servicebio), F4/80 (1:5000, batch number: GB113373, Servicebio), or CD 31 (1:200, batch number: GB 15063, Servicebio) in 1% BSA solution. After washing, the corresponding HRP-conjugated secondary antibodies were applied, and the sections were incubated at 37 °C for 50 min. Finally, an appropriate amount of antifade reagent (including DAPI) was added, and the sections were incubated for 10 min. Fluorescent images were captured using a fluorescence microscope(Nikon ECLIPSE E100, Nikon Corporation, Tokyo, Japan).

#### 4.4.7. Immunohistochemistry Analysis

The procedure for immunohistochemistry analysis was similar to that for immunofluorescence staining. First, tissue sections were deparaffinized and rehydrated, followed by treatment with 3% hydrogen peroxide solution to block endogenous peroxidase activity. The sections were then washed three times with PBS and blocked with 3% BSA at room temperature for 30 min. Subsequently, the sections were incubated overnight at 4 °C with primary antibodies against VEGF (1:200, batch number: GB 15165, Servicebio), Ki-67 (1:500, batch number: GB 111141, Servicebio), TNF-α (1:500, batch number: GB 115702, Servicebio), or TGF-β (1:200, batch number: GB 11179, Servicebio). After washing, the corresponding secondary antibodies were applied, and the sections were incubated further. Finally, the sections were developed with freshly prepared DAB chromogenic solution, counterstained with hematoxylin, dehydrated, and mounted. The stained sections were observed under a bright-field microscope, and images were collected.

#### 4.4.8. ELISA Detection

Blood samples were collected from the eyeballs of the mice. After standing for 2 h, the samples were centrifuged at −4 °C and 10,000 rpm for 15 min using a Velocity 18R centrifuge (Dynamica Scientific Ltd., London, UK). The supernatant was collected for analysis. The concentrations of IL-6 (catalog number: 388298, Thermo Fisher Scientific, Waltham, MA, USA), IL-1β (catalog number: 383584-002, Thermo Fisher Scientific, Waltham, MA, USA), IL-10 (catalog number: 375943-003, Thermo Fisher Scientific, Waltham, MA, USA), and IL-2 (catalog number: 397851-002, Thermo Fisher Scientific, Waltham, MA, USA) were determined using ELISA kits. All samples were analyzed on an FRT6100 microplate reader (Redou Life Science Co., Ltd., Shenzhen, China), and the assay procedures were strictly followed as per the manufacturer’s instructions.

#### 4.4.9. Measurement of Oxidative Damage

The level of malondialdehyde (MDA) in serum was measured using an MDA assay kit (catalog number: MPC2405084, Servicebio). The measurements were performed using an Epoch microplate reader (BioTek Instruments, Winusky, VT, USA). All procedures were carried out according to the manufacturer’s instructions.

### 4.5. Hydrogel Toxicity Assessment

On day 20 post-experiment, to ensure the welfare of the mice, the animals were euthanized humanely. Their livers were then collected for histological analysis. After routine tissue processing, liver sections were prepared and stained with hematoxylin and eosin (H&E). The morphology of the tissues was examined using a NIKON ECLIPSE E100 optical microscope to assess any potential toxicity of the hydrogel.

### 4.6. Statistical Analysis

All experimental data were analyzed using GraphPad Prism 9.0 software. Data are presented as mean ± standard deviation (Mean ± SD). Intergroup comparisons were performed using one-way analysis of variance (ANOVA), followed by Tukey’s HSD test for multiple comparisons. A *p*-value of less than 0.05 was considered statistically significant.

## Figures and Tables

**Table 1:** Potential targets of B-CGT hydrogel.

Target Name	Uniprot_ID	Protein	Probability
SGLT2	P31639	Sodium/glucose cotransporter 2	1
SGLT1	P13866	Sodium/glucose cotransporter 1	1
PTPN1	P18031	Tyrosine-protein phosphatase non-receptor type 1	0.999
GALR3	O60755	Galanin receptor type 3	0.979
TOP1	P11387	DNA topoisomerase 1	0.921
MMP12	P39900	Macrophage metalloelastase	0.891
PPO2	O42713	Polyphenol oxidase 2	0.855
ALR2	P07943	Aldose reductase	0.845
CA13	Q8N1Q1	Carbonic anhydrase 13	0.733
SIRT1	Q96EB6	NAD-dependent protein deacetylase sirtuin-1	0.725
CASP8	Q14790	Caspase-8	0.531
MMP7	P09237	Matrilysin	0.422
CA14	Q9ULX7	Carbonic anhydrase 14	0.327
CA12	O43570	Carbonic anhydrase 12	0.118
FFAR1	O14842	Free fatty acid receptor 1	0.114
PTGS1	P05979	Prostaglandin G/H synthase 1	0.105
CTSK	P43235	Cathepsin K	0.097
NR2F2	P24468	COUP transcription factor 2	0.095
ADORA1	P25099	Adenosine receptor A1	0.061
BCL2A1	Q16548	Bcl-2-related protein A1	0.045
DNMT1	P26358	DNA (cytosine-5)-methyltransferase 1	0.024
MME	P07861	Neprilysin	0.022
ADORA3	P28647	Adenosine receptor A3	0.009
CDC25B	P30305	M-phase inducer phosphatase 2	0.009
EDNRB	P21451	Endothelin B receptor	0.006
NTSR1	P30989	Neurotensin receptor type 1	0.006
CASP1	P29466	Caspase-1	0.004
CACNA1B	Q00975	Voltage-dependent N-type calcium channel subunit alpha-1B	0.003
Lethal factor	P15917	Lethal factor	0.003
CASP9	P55211	Caspase-9	0.001
GPR35	Q9HC97	G-protein coupled receptor 35	0.001
MMP8	P22894	Neutrophil collagenase	0.001

**Table 2:** Information on the degree centrality, betweenness centrality, closeness centrality, eigenvector centrality, network centrality, and local average connectivity of core targets.

Target Name	DC	EC	LAC	BC	CC	NC
SIRT1	14	0.32	8.57	20.55	0.32	13.36
CASP8	13	0.31	8.92	8.42	0.32	12.40
CASP9	13	0.31	8.92	8.42	0.32	12.40
CASP1	12	0.30	9.00	4.15	0.31	11.39
DNMT1	12	0.28	7.17	14.32	0.31	9.85
PTGS1	12	0.27	6.83	43.25	0.31	8.24
MMP8	10	0.26	7.80	2.48	0.30	9.03

**Table 3:** GO enrichment analysis of drug–disease intersection targets (top five entries with significant *p*-values across CC, BP, and MF categories).

Classification	GO ID	Description	*p*-Value	*p*.Adjust	Genes
CC	GO:0044297	cell body	0.0001730	0.0120507	CASP8; NTSR1; ADORA1; MME; TOP1
CC	GO:0045121	Membrane raft	0.0002371	0.0120507	CASP8; NTSR1; EDNRB; MME
CC	GO:0044306	Neuron projection terminus	0.0002373	0.0120507	NTSR1; ADORA1; MME
CC	GO:0098857	Membrane microdomain	0.0002398	0.0120507	CASP8; NTSR1; EDNRB; MME
CC	GO:0031904	Endosome lumen	0.0006352	0.0255344	CTSK; PTPN1
BP	GO:1901216	Positive regulation of neuron death	0.0001011	0.0032803	CASP8; CASP9; ADORA1
BP	GO:0016485	Protein processing	0.0001029	0.0032803	CASP8; CASP9; CASP1; MME
BP	GO:0034644	Cellular response to UV	0.0001079	0.0033788	SIRT1; CASP9; MME
BP	GO:0048522	Positive regulation of cellular process	0.0001142	0.0035093	CASP8; SIRT1; NTSR1; MMP8; DNMT1; CASP9; EDNRB; MMP7; CASP1; ADORA1; GPR35; NR2F2; PTPN1; MME
BP	GO:0055089	Fatty acid homeostasis	0.0001160	0.0035093	SIRT1; ADORA1
MF	GO:0016787	Hydrolase activity	0.0002149	0.0043864	CASP8; SIRT1; MMP8; CASP9; MMP7; CASP1; CTSK; PTPN1; MME
MF	GO:0004222	Metalloendopeptidase activity	0.0002354	0.0043864	MMP8; MMP7; MME
MF	GO:0008528	G protein-coupled peptide receptor activity	0.0003557	0.0059849	NTSR1; EDNRB; GPR35
MF	GO:0001653	Peptide receptor activity	0.0004006	0.0059849	NTSR1; EDNRB; GPR35
MF	GO:0019904	Protein domain specific binding	0.0004087	0.0059849	CASP8; SIRT1; CASP9; CASP1; TOP1

**Table 4:** KEGG enrichment analysis of drug–disease intersection targets (including the Top 30 pathways ranked by significance based on *p*-value).

KEGG_A_Class	KEGG_B_Class	Pathway	*p*-Value	Genes	Ratio
Human Diseases	Infectious disease: bacterial	Legionellosis	0.00014	CASP8/CASP9/CASP1	0.052
Cellular Processes	Cell growth and death	Apoptosis—multiple species	0.00144	CASP8/CASP9	0.062
Environmental Information Processing	Signaling molecules and interaction	Neuroactive ligand–receptor interaction	0.00328	NTSR1/EDNRB/ADORA1/GPR35	0.011
Human Diseases	Infectious disease: viral	Influenza A	0.00351	CASP8/CASP9/CASP1	0.017
Organismal Systems	Endocrine system	Regulation of lipolysis in adipocytes	0.00549	PTGS1/ADORA1	0.032
Human Diseases	Cardiovascular disease	Lipid and atherosclerosis	0.00653	CASP8/CASP9/CASP1	0.014
Human Diseases	Drug resistance: antineoplastic	Platinum drug resistance	0.00732	CASP8/CASP9	0.027
Cellular Processes	Cell growth and death	p53 signaling pathway	0.00732	CASP8/CASP9	0.027
Human Diseases	Infectious disease: bacterial	Pathogenic Escherichia coli infection	0.01261	CASP8/CASP9/CASP1	0.011
Organismal Systems	Immune system	Toll-like receptor signaling pathway	0.01498	CASP8/CTSK	0.019
Organismal Systems	Immune system	C-type lectin receptor signaling pathway	0.01607	CASP8/CASP1	0.018
Human Diseases	Infectious disease: parasitic	Toxoplasmosis	0.01719	CASP8/CASP9	0.018
Human Diseases	Cardiovascular disease	Viral myocarditis	0.02297	CASP8/CASP9	0.015
Human Diseases	Endocrine and metabolic disease	Alcoholic liver disease	0.02733	CASP8/SIRT1	0.014
Human Diseases	Infectious disease: viral	Measles	0.02839	CASP8/CASP9	0.013
Human Diseases	Infectious disease: viral	Hepatitis C	0.03313	CASP8/CASP9	0.012
Human Diseases	Neurodegenerative disease	Alzheimer disease	0.03441	CASP8/CASP9/MME	0.007
Human Diseases	Infectious disease: viral	Hepatitis B	0.03465	CASP8/CASP9	0.012
Cellular Processes	Cell growth and death	Necroptosis	0.03503	CASP8/CASP1	0.012
Human Diseases	Cancer: overview	MicroRNAs in cancer	0.03776	SIRT1/DNMT1	0.011
Environmental Information Processing	Signal transduction	cGMP-PKG signaling pathway	0.03816	EDNRB/ADORA1	0.011
Organismal Systems	Endocrine system	Renin–angiotensin system	0.04218	MME	0.042
Organismal Systems	Immune system	NOD-like receptor signaling pathway	0.04432	CASP8/CASP1	0.011
Human Diseases	Infectious disease: viral	Kaposi sarcoma-associated herpesvirus infection	0.04818	CASP8/CASP9	0.01
Human Diseases	Infectious disease: viral	Human immunodeficiency virus 1 infection	0.05906	CASP8/CASP9	0.009
Metabolism	Metabolism of cofactors and vitamins	Nicotinate and nicotinamide metabolism	0.06265	SIRT1	0.028
Human Diseases	Infectious disease: viral	Human cytomegalovirus infection	0.06432	CASP8/CASP9	0.009
Organismal Systems	Immune system	Toll and Imd signaling pathway	0.06938	CASP8	0.025
Human Diseases	Cancer: overview	Pathways in cancer	0.06981	CASP8/CASP9/EDNRB	0.005
Human Diseases	Infectious disease: bacterial	Tuberculosis	0.07683	CASP8/CASP9	0.008

**Table 5:** Molecular docking results.

Target Name	Total Score	Crash	Polar
SIRT1	10.0932	−2.6568	4.3866
CASP8	4.9651	−1.4622	4.4537
CASP9	7.8708	−2.5205	5.8399
CASP1	4.9301	−1.5732	1.0869
DNMT1	7.742	−1.3801	4.8213
PTGS1	7.8977	−2.8520	5.7447
MMP8	7.9809	−2.8087	3.9987

## Data Availability

Data will be made available on request.

## Abbreviations

B-CGT: Hydrogel of Glucosyloxybenzyl 2-Isobutylmalates from Bletilla striata; TNF-α, Tumor Necrosis Factor-alpha; IL-1β, Interleukin-1 beta; IL-6, Interleukin-6; IL-10, Interleukin-10; TGF-β, Transforming Growth Factor Beta; VEGF, Vascular Endothelial Growth Factor; ECM, extracellular matrix; CG, control group; MG, model group; PC, positive control group; H&E, Hematoxylin and Eosin; MDA, Malondialdehyde; DC, degree centrality; BC, betweenness centrality; CC, closeness centrality; EC, eigenvector centrality; NC, network centrality; LAC, local average connectivity; GO, Gene Ontology; BP, biological processes; MF, molecular functions.
